# Successful Conversion of the *Bacillus subtilis* BirA Group II Biotin Protein Ligase into a Group I Ligase

**DOI:** 10.1371/journal.pone.0096757

**Published:** 2014-05-09

**Authors:** Sarah K. Henke, John E. Cronan

**Affiliations:** 1 Department of Microbiology, University of Illinois, Urbana, Illinois, United States of America; 2 Department of Biochemistry, University of Illinois, Urbana, Illinois, United States of America; University of Florida, United States of America

## Abstract

Group II biotin protein ligases (BPLs) are characterized by the presence of an N-terminal DNA binding domain that allows transcriptional regulation of biotin biosynthetic and transport genes whereas Group I BPLs lack this N-terminal domain. The *Bacillus subtilis* BPL, BirA, is classified as a Group II BPL based on sequence predictions of an N-terminal helix-turn-helix motif and mutational alteration of its regulatory properties. We report evidence that *B. subtilis* BirA is a Group II BPL that regulates transcription at three genomic sites: *bioWAFDBI*, *yuiG* and *yhfUTS*. Moreover, unlike the paradigm Group II BPL, *E. coli* BirA, the N-terminal DNA binding domain can be deleted from *Bacillus subtilis* BirA without adverse effects on its ligase function. This is the first example of successful conversion of a Group II BPL to a Group I BPL with retention of full ligase activity.

## Introduction

**Figure 1:**
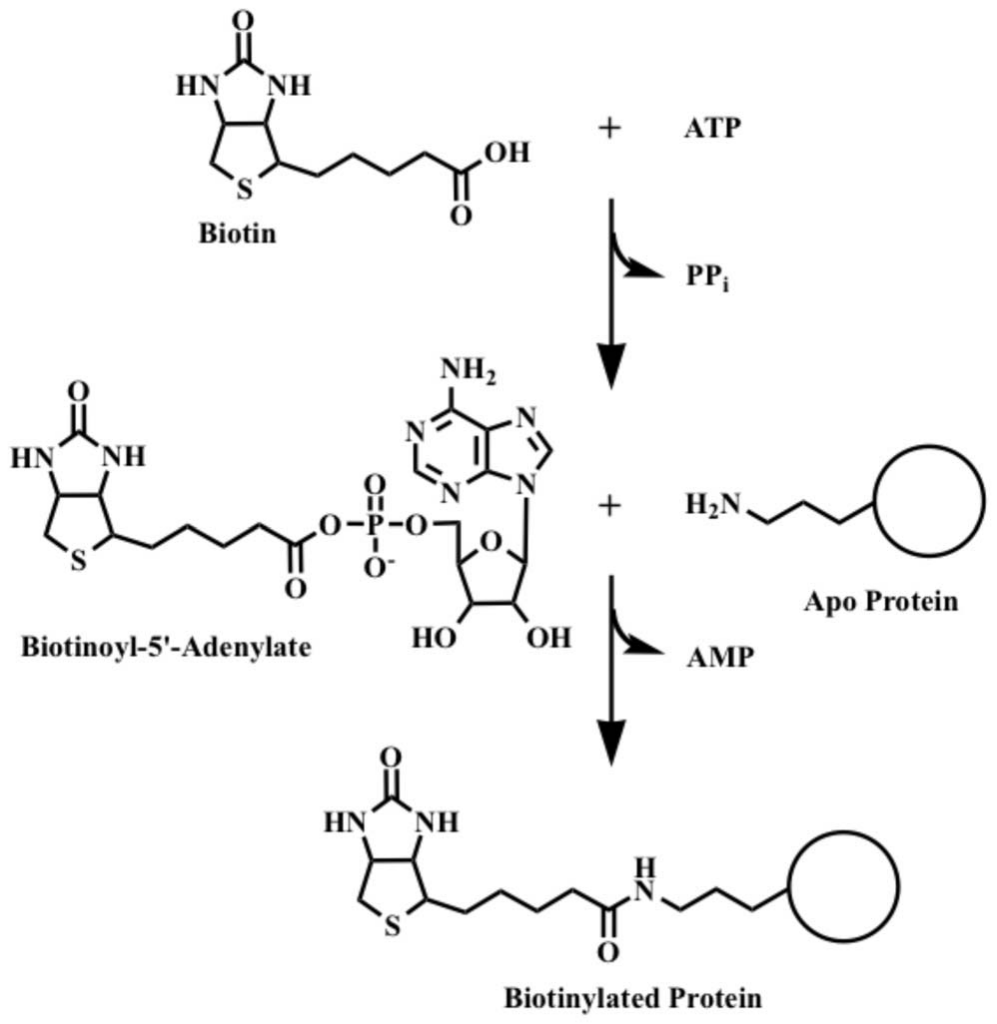
The biotin protein ligase (BPL) reaction. Attachment of biotin to acceptor proteins occurs in a two-step reaction. First, BirA binds biotin and ATP to synthesize Bio-5′-AMP (biotinoyl-5'-adenylate) with release of pyrophosphate. In the second step the conserved lysine residue of the acceptor protein acts as a nucleophile and attacks the mixed anhydride bond to give the biotinylated acceptor protein plus AMP.

Biotin protein ligase (BPL) is required for the covalent attachment of biotin to biotin-dependent enzymes. This attachment proceeds in a two-step reaction. First, BPL binds both biotin and ATP to synthesize biotinoyl-5′-AMP (Bio-5′-AMP, also called biotinoyl-adenylate) with release of pyrophosphate [1]. The ε-amino group of the conserved lysine residue of the acceptor protein acts as a nucleophile to attack the Bio-5′-AMP mixed anhydride bond to give covalently attached biotin plus AMP (Fig. 1). Microbial BPLs are readily placed into two groups [2]. Both groups have catalytic and C-terminal domains that show strong structural conservation [3]–[7] whereas Group II BPLs are characterized by addition of an N-terminal helix-turn-helix (HTH) DNA binding domain that permits transcriptional regulation of the biotin synthetic genes. *E. coli* BirA, the paradigm for regulation of biotin biosynthesis, is the best studied Group II BPL. Transcriptional repression of the *E. coli* biotin operon occurs when BirA accumulates Bio-5′-AMP because all biotin acceptor proteins have been biotinylated [8]–[10]. Bio-5′-AMP accumulation results in dimerization of BirA and subsequent DNA binding [6], [11], [12]. In all four *E. coli* BirA crystal structures [13]–[15] the HTH structure is spatially well removed from the other domains of the protein and thus deletion of the N-terminal DNA binding domain was expected to convert this Group II BPL into a fully functional Group I ligase. However, this was not the case: the resulting protein had severely compromised ligase activity [16]. This was also true for ligases having smaller N-terminal deletions [17].

**Figure 2:**
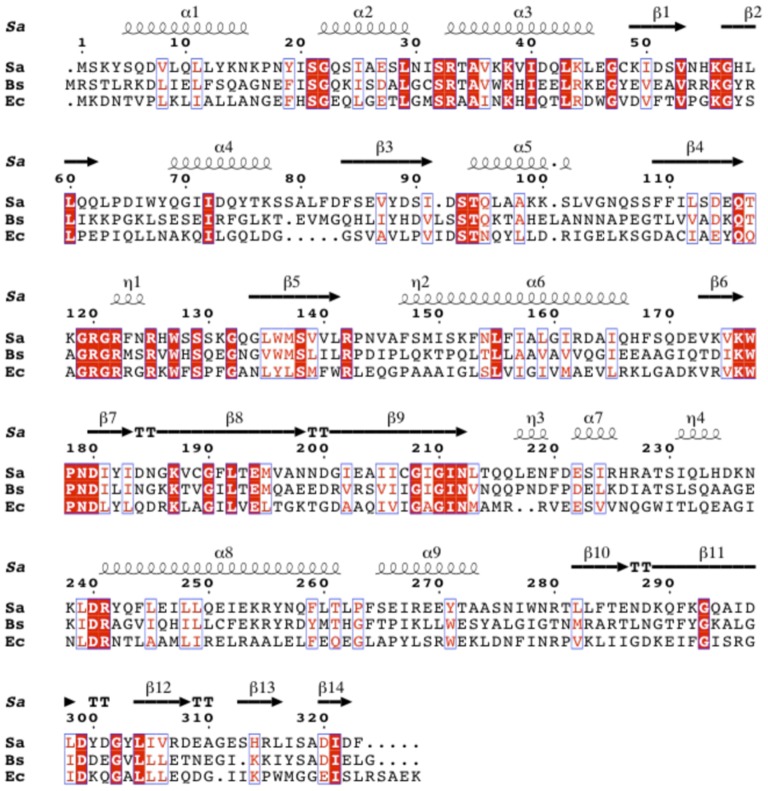
Sequence alignments of *S. aureus* BPL, *B. subtilis* BirA and *E. coli* BirA. *B. subtilis* BirA has 31% amino acid identity to *S. aureus* BPL and 27% amino acid identity to *E. coli* BirA. Conserved residues are in white text and highlighted in red and similar residues are in red text and boxed in blue. The *S. aureus* BPL secondary structure (PDB: 4DQ2) is shown above the amino acid sequence.

The sequences of other Group II BPLs suggest such proteins are found in γ-Proteobacteria, Bacilli, and Clostridii [2], although only the proteins from *E. coli* [6] and (very recently) *Staphylococcus aureus* [7] have been enzymatically characterized and crystallized. One of the Group II BPLs, *B. subtilis* BirA, has only 27% amino acid sequence identity to *E. coli* BirA (Fig. 2). Despite this low sequence identity, *Bacillus subtilis birA* has been shown to complement the ligase activity of a temperature-sensitive *E. coli birA85* strain [18]. Moreover, *B. subtilis birA* mutants show constitutive expression of a *bioW-lacZ* fusion, suggesting that BirA regulates biotin operon transcription [18]. *B. subtilis* microarray data identified two additional transcripts, *yuiG* and *yhfUST*, regulated by biotin and BirA [19]. Both YuiG and YhfU have strong sequence similarity to the structurally characterized BioY biotin transporter of *Lactococcus lactis* [20] and other well characterized energy-coupling factor (ECF) biotin transporters [21]. All three transcripts have similar predicted BirA binding sites [2], [19]. *B. subtilis* has two known biotinylated proteins, pyruvate carboxylase (PyC) and the biotin carboxyl carrier protein (AccB) subunit of acetyl-CoA carboxylase (http://genodb.pasteur.fr). A third *B. subtilis* protein, biotin/lipoyl attachment protein (BLAP) encoded by the *yngHB* gene was found to be biotinylated and lipoylated when expressed in *E. coli* [22] although subsequently this protein was found not to be lipoylated by the *B. subtilis* enzymes that modify the known cognate lipoic acid acceptor proteins [23].

A major shortcoming of our picture of the enzymatic and *in vivo* regulatory activities of Group II BPLs is that it is based on a single example, *E. coli* BirA. For this reason we decided to study the enzymatic and regulatory properties of the BirA of *B. subtilis*, a bacterium that is evolutionarily diverse from *E. coli.* Moreover the amino acid sequence of *B. subtilis* BirA differs markedly from that of the *E. coli* protein and *in vivo B. subtilis* BirA biotinylates multiple proteins whereas *E. coli* BirA modifies only a single protein. Here we report that *B. subtilis* BirA is a Group II BPL that follows the *E. coli* model of regulation, but unlike the *E. coli* protein, *B. subtilis* BirA can be successfully converted into a fully active Group I BPL.

## Materials and Methods

### Strains chemicals and culture media

The bacterial strains used were derivatives of *B. subtilis* 168 and *E. coli* K-12 (Table 1). The rich medium used to grow *E. coli* and *B. subtilis* was LB. The defined medium for *E. coli* was M9 salts supplemented with 0.5% glucose and 0.01% vitamin-free Casamino Acids (Difco) whereas the defined medium for *B. subtilis* was Spizizen salts [24] supplemented with 0.5% glycerol and 0.05% vitamin-free Casamino Acids (Difco) in addition to 0.01% each of tryptophan, tyrosine, isoleucine and phenylalanine. Antibiotics were used at the following concentrations (in µg ml^−1^): sodium ampicillin, 100; kanamycin sulfate, 50; chloramphenicol, 25; tetracycline HCl, 12; erythromycin, 100; lincomycin, 12.5; streptomycin sulfate, 50 and spectinomycin sulfate, 50. The 15∶1 mixture of ticarcillin disodium salt and potassium clavulanate (Research Products International) was used at 25 µg ml^−1^. Oligonucleotides were purchased from Integrated DNA Technologies. PCR amplification was performed using Taq polymerase (New England BioLabs) and *Pfu* polymerase (Stratagene) according to the manufacturer's specifications. DNA constructs were sequenced by ACGT, Inc. Reagents and chemicals were obtained from Sigma-Aldrich and Fisher, unless otherwise noted. New England BioLabs supplied restriction enzymes and T4 DNA ligase. Life Technologies provided SYBER Green I Nucleic Acid Gel stain and the 6% DNA Retardation Novex TBE Gels. Perkin Elmer provided [α-^32^P]ATP (6,000 Ci/mmol). Analtech TLC Uniplates of microcrystalline cellulose matrix were purchased from Sigma-Aldrich.

**Table 1:** Bacterial strains.

Strain	Relevant Genotype or Description	Reference or Derivation
*B. subtilis*		
168	*trpC2*	Lab collection
1A330	*aroG932 bioB141*	BGSC
SKH001	1A330 *bioW::lacZ*	This study
SKH002	SKH001 *amyE*:: P spac *accB-86*	This study
*E. coli*		
MG1655	*E. coli* K-12 wild type	Lab collection
DH5α	*Δ(argF-lacZ)U169 glnV44 Φ80 Δ(lacZ)M15 gyrA96 recA1 relA1 endA1 thi-1 hsdR17*	Lab collection
BM4092	[*araD139]B/r, Δ(argF-lac)169, λ-, TP(bioF-lacZ)501, flhD5301, Δ(fruK-yeiR)725(fruA25), relA1, rpsL150(strR), rbsR22, birA1, Δ(fimB-fimE)632(::IS1), deoC1*	[33]
BL21 λ (DE3)	*ompT, hsdSB (rB–, mB–), dcm, gal, λ(DE3)*	Novagen
VC618	MG1655 *Δ lacZY, bioF::lacZY, birA::Km^R^,* pVC18	[36]
VC832	BL21 λ (DE3), pVC36	[17]

**Table 2:** Plasmids.

Plasmid	Relevent Genotype or Description	Reference or Derivation
pET19b	T7 promoter expression vector, AmpR	Novagen
pSKH001	pET19b Encoding N-terminal hexahistidine tagged *B. subtilis* BirA, AmpR	This study
pET28b	T7 promoter expression vector, KanR	Novagen
pSKH003	pET28 encoding accB-86	This study
pSKH004	pET28 encoding pyc-77	This study
pQC026	pET28b encoding C-terminal Hexahistidine tagged BLAP	[23]
pBAD322K	Medium copy expression vector, KanR	[48]
pSKH005	pBAD322K encoding *B. subtilis* 64-325	This study
pSKH006	pBAD322K encoding *B. subtilis* WT BirA	This study
pSKH007	pBAD322K encoding *E. coli* BirA 66-321	This study
pSKH008	pBAD322K encoding *E. coli* WT BirA	This study
pSKH009	pBAD322K encoding *B. subtilis* 66-325	This study
pSKH010	pBAD322K encoding *B. subtilis* 75-325	This study
pSKH011	pBAD322K encoding *B. subtilis* 82-325	This study
pBAD322Cm	Medium copy expression vector, CmR	[48]
pSKH012	pBAD322Cm encoding *B. subtilis* 64-325	This study
pSKH013	pBAD322Cm encoding *B. subtilis* WT BirA	This study
pSKH014	pBAD322Cm encoding *E. coli* BirA 66-321	This study
pSKH015	pBAD322Cm encoding *E. coli* WT BirA	This study
pSKH016	pBAD322Cm encoding *B. subtilis* 66-325	This study
pSKH017	pBAD322Cm encoding *B. subtilis* 75-325	This study
pSKH018	pBAD322Cm encoding *B. subtilis* 82-325	This study
pSKH019	pET19b encoding N-terminal hexahistidine tagged BirA 64-325, AmpR	This study
pSKH020	pET19b encoding N-terminal hexahistidine tagged BirA 66-325, AmpR	This study
pSKH021	pET19b encoding N-terminal hexahistidine tagged BirA 75-325, AmpR	This study
pSKH022	pET19b encoding N-terminal hexahistidine tagged BirA 82-325, AmpR	This study
pMUTIN4	spoVG-*lacZ*, ermR	[49]
pDR111	SpcR, 5'-*amyE*, 3'-*amyE*, p*hyper-spank*	G. W. Ordal
pSKH023	pMUTIN4 encoding *B. subtilis bioW* internal 500 bp fragment	This study
pSKH024	pDR111 encoding *B. subtilis accB-86*	This study

### Plasmids and plasmid constructions

The plasmids used and constructed are given in Table. 2. The *B. subtilis birA* gene was amplified by PCR from *B. subtilis* strain 168 genomic DNA with primers SKH001 and SKH002 (all primers are given in Table 3) that added NdeI and XhoI sites. The product was digested with NdeI and XhoI and ligated into the same sites of pET19b, with an N-terminal hexahistidine tag to give pSKH001.

**Table 3:** Oligonucleotides utilized.

Oligo-nucleotide	Description	Sequence
SKH001	BirA F NdeI	GAGTGGCTGAACATATGCGGTCAAC
SKH002	BirA stop R Xhol	GGCTTGTACCCTCGAGTTAGCCCAATTC
SKH005	accB F NcoI	CCATGGAAAAGCAAGATGAGAATCTGCATAAA
SKH006	accB stop R XhoI	CTCGAGCTTACTCCGCTTTTACAAGAAATAGAGG
SKH007	pyc F NcoI	CCATGGAACGGACAAATCCAAGCCAC
SKH009	pyc stop R XhoI	CTCGAGTTTATGCTTTTTCAATTTCAAGGAGC
SKH014	bioO F	GATCCTTTCTTCTATTGACAGAAAC
SKH015	bioO R	CGC CCTTTCACTGATAACTGAAGAAC
SKH016	yhfU F	ACCATCAAAAACCGGTCTGCCATAC
SKH017	yhfU R	CCAAAAAGTAATCAAATATGGTTATAC
SKH018	yuiG F	ATTGATCGGACTGTCTTGTT
SKH019	yuiG R	CCCTTAGGTTGACATACACA
SKH026	EC bioO F	GATATGGCGTTGGTCAAAGGCAAG
SKH027	EC bioO R	GGGGCTTCTCCAAAACGTGTTTTTTG
SKH034	bioO half F	GAAAAAGACCGTTTTGTGTG
SKH035	bioO half R	ATTCAAAGGTTAACAATTAGAATATATTATTCTCTCCTG
SKH028	Non 125 F	GCATGACGGTTAGCATACAAATGGCAG
SKH029	Non 125 R	AACGATCGGGATTTCCATTTTCATCGATTC
SKH036	BirA 64-325 EcoRI F	ACTGCGAATTCACCATGAAACCCGGAAAACTCAGTGAAAGCG
SKH037	BirA SalI R	ACTGAGTCGACTTAGCCCAATTCGATATCGGCAG
SKH038	BirA EcoRI F	CTGACGAATTCACCATGCGGTCAACATTAAGAAAAGACC
SKH040	BirA 64-325 NdeI F	CAGTCCATATGAAACCCGGAAAACTCAGTGAAAG
SKH041	BirA BamHI R	ACTGGGATCCTTAGCCCAATTCGATATCG
SKH042	EC BirA 65-321 EcoRI F	ACTGCGAATTCACCATGCAGTTACTTAATGCTAAACAGATATTG
SKH043	EC BirA SalI R	CAGTCGTCGACTTATTTTTCTGCACTACGCAG
SKH044	BirA 82-325 NdeI F	ACGTACATATGGGCCAGCATCTTATTTACCATG
SKH045	BirA 82-325 EcoRI F	AGCTAGAATTCACCATGGGCCAGCATCTTATTTAC
SKH046	BirA 66-325 EcoRI F	ACTGCGAATTCACCATGGGAAAACTCAGTGAAAGCG
SKH047	BirA 66-325 NdeI F	CAGTCCATATGGGAAAACTCAGTGAAAGCG
SKH048	BirA 75-325 EcoRI F	ACTGCGAATTCACCATGTTTGGATTAAAAACGGAAG
SKH049	EC BirA EcoRI F	ACTGGAATTCACCATGAAGGATAACACCGTGCCAC
SKH050	BirA 75-325 NdeI F	CAGTCCATATGTTTGGATTAAAAACGGAAG
SKH057	bioW 500 F	ACGTGAATTCCATACAGTCAATGCTTTATTAG
SKH058	bioW 500 R	GACTGGATCCCTTACCCGCAACATAGCCTG
SKH065	ycgB F	CTTACAGAAGAGCGGTAAAAGAAGAAATAAAAAAG
SKH066	lacI R	CCGTCTCACTGGTGAAAAGAAAAAC
SKH067	ldH R	CATTGCTTTTTCTTTATTTACATCAATGACCACAA
SKH069	RBS accb-86 F	ACGGTCGACAAGGAGGAAAAAATATGGAAGCACCAAAGCAAGATG
SKH070	accb-86 R	GACGCATGCTTACTCCGCTTTTACAAGAAATAGAGGTTGTC
SKH073	pDR111 F Seq	CTCGAGGGTAAATGTGAGCACTCAC
SKH074	pDR111 R Seq	GAAAGTATTACATATGTAAGATTTAAATGCAACCG
SKH075	specR F	TGAATCTTCTCCATTAGAACATAGGGAGAG
MM1	5' BioW F	CGA TCC TTT CTT CTA TTG ACA GAA ACA GG
MM2	LacZ R	GGT GTA GAT GGG CGC ATC GTA AC
MM3	pSPAC F	CTA CAC AGC CCA GTC CAG ACT ATT CGG
MM4	3' BioW R	ATG GCG TCA TCT AGT TCT TTT TTG CGG

The C-terminal 86 residues of *B. subtilis* AccB (AccB-86) were amplified by PCR from *B. subtilis* strain 168 genomic DNA with primers SKH005 and SKH006 that added NcoI and XhoI sites. The product was digested with NcoI and XhoI and ligated into the same sites of pET-28 to give pSKH003 which encoded the untagged protein. The C-terminal 77 residues of *B. subtilis* PyC (PyC-77) were amplified by PCR from *B. subtilis* strain 168 genomic DNA with primers SKH007 and SKH009 and inserted into pET-28 by the same procedures.

*B. subtilis* BirA, Δ2-64 BirA, Δ2-66 BirA, Δ2-74 BirA, and Δ1-81 BirA were amplified by PCR from *B. subtilis* strain 168 genomic DNA with forward primers SKH038, SKH036, SKH046, SKH048, SKH045, respectively, plus reverse primer SKH037. The primers added EcoRI and SalI sites. The products were digested with EcoRI and SalI and ligated into the same sites of pBAD322K, to give pSKH006, pSKH005, pSKH009, pSKH010 and pSKH011, respectively. The products were also ligated into the same sites of pBAD322Cm to give pSKH013, pSKH012, pSKH016, pSKH017 and pAKH018, respectively. *E. coli* BirA and Δ2-64 BirA were amplified by PCR from *E. coli* MG1655 genomic DNA with primers SKH049 and SKH043, and SKH042 and SKH043, respectively, thereby adding EcoRI and SalI sites. The products were digested with EcoRI and SalI and ligated into the same sites of pBAD322K, to give pSKH008 and pSKH007 respectively. The products were also ligated into the same sites of pBAD322Cm to give pSKH015 and pSKH014 respectively.

*B. subtilis* Δ2-64 BirA, Δ2-66 BirA, Δ2-74 BirA, and Δ1-81 BirA were amplified by PCR from *B. subtilis* strain 168 genomic DNA with forward primers SKH040, SKH047, SKH050, SKH044, respectively plus reverse primer SKH041. The primers added NdeI and XhoI sites. The products were digested with NdeI and XhoI and ligated into the same sites of pET-19b, adding an N-terminal hexahistidine-tag, to give pSKH019, pSKH020, pSKH021, and pSKH022, respectively.

A 500 base pair internal fragment of *B. subtilis bioW* was PCR amplified from *B. subtilis* strain 168 genomic DNA with primers SKH057 and SKH058 that added EcoRI and BamHI sites. The product was digested with EcoRI and BamHI and ligated into the same sites of pMUTIN4 to give pSKH023.

*B. subtilis accB-86* was PCR amplified from strain 168 genomic DNA with primer SKH069 which added a HindIII and a strong RBS site (AAGGAGGAAAAAATATG) plus primer SKH070 which contained an SphI site. The product was digested with HindIII and SphI and ligated into the same sites of pDR111 to give pSKH0024.

### *Bacillus subtilis* strain construction

*B. subtilis* competent cell preparation and transformation were carried out as described by Dubnau and Davidoff-Abelson [25]. To create a *bioW-lacZ* chromosomal fusion, pSKH023 was transformed into strain 1A330 creating strain SKH001. Single crossover integration into *bioW* was verified by PCR and sequencing. To construct an IPTG inducible chromosomal copy of *accB-86*, strain SKH001 was transformed with linearized pSKH024 creating strain SKH002. Double crossover integration into *amyE* was verified by the amylase production screen of Harwood and Cutting [26]. Integration was also verified by PCR and by sequencing with primers SKH065 and SKH066, SKH067 and SKH075 and SKH073 and SKH074.

### Structural modeling and sequence alignment

The *B. subtilis* AccB biotin attachment domain was identified by InterProScan [27] and structural modeling to *E. coli* AccB-87 crystal structure (PDB 1A6X) using Swiss-Model automated mode [28]-[30]. The *B. subtilis* PyC biotin attachment domain was identified by InterProScan [27] and structural modeling to the *Staphylococcus aureus* pyruvate carboxylase biotin attachment domain crystal structure (3HBL) using Swiss-Model as above. *B. subtilis* BirA N-terminal deletions were determined by modeling to *S. aureus* BirA crystal structure (4DQ2) as a template using Swiss-Model automated mode as above. The final image was created using UCSF Chimera package [31]. Sequence alignments were created using the Clustal Omega (http://www.ebi.ac.uk/Tools/msa/clustalo/) and the output was processed by ESPript 3.0 (http://espript.ibcp.fr/ESPript/cgi-bin/ESPript.cgi) to generate the final figure [32].

### Complementation analyses

Two *E. coli birA* strains were tested. In the more straightforward test strain BM4092 [33] was transformed with either plasmid pSKH001 or pSKH002 followed by selection for transformants by plating on LB supplemented with ampicillin or kanamycin, respectively. These strains were grown at 37°C on M9 minimal plates [34] with 25 µM 5-bromo-4-chloro-indolyl-β-D-galactopyranoside (X-gal) and varying concentrations of biotin (1.6 nM, 4.1 nM, 41 nM, 4.1 µM, or 41 µM) [33]. Note that gene expression from T7 promoter based multi-copy plasmids in the absence of T7 RNA polymerase has been shown to be equivalent to that of a single copy plasmid [35]. Derivatives of strain BM4092 expressing the mutant BirAs encoded by kanamycin resistant plasmids pSKH005, pSKH006, pSKH007, pSKH008, pSKH009, pSKH010 or pSKH011 were similarly obtained and tested.

Strain VC618 which carries a deletion of the chromosomal *birA* gene was transformed with pSKH012, pSKH013, pSKH014, pSKH015, pAKH016, pSKH017, or pSKH018 and transformants were selected on LB plates supplemented with ampicillin and chloramphenicol at 30°C. These strains were cured of the temperature sensitive plasmid VC18 which expresses the *Saccharomyces cerevisiae* Bpl1 ligase by growth at 42°C on the defined medium supplemented with chloramphenicol and biotin. Loss of the temperature-sensitive plasmid was indicated by ampicillin sensitivity [36]. These strains were then grown at 37°C on M9 minimal plates [34] with 25 µM X-gal and varying concentrations of biotin as above [33].

### Protein purification

For purification of the wild type and *B. subtilis* N-terminally deleted BirA Proteins *E. coli* strain BL21 (λ DE3) was transformed with pSKH001, pSKH019, pSKH020, pSKH021, or pSKH022. The strains were grown at 37°C in LB medium supplemented with ticarcillin-clavulanate to an OD_600_ of 0.8 and induced by addition of IPTG to 1 mM for an additional 6 h at 37°C. Cells were centrifuged and resuspended in lysis buffer which was 50 mM Tris-HCl, 500 mM NaCl, 0.1 mM tris(2-carboxyethyl)phosphine (TCEP), 10 mM imidazole, 5% glycerol, pH 8.0). The cells were lysed by passage through a French pressure cell and the lysate was centrifuged. The supernatant was added to Ni NTA beads (Qiagen) and incubated for 30 min. The mixture was added to a disposable 10 ml polypropylene column (Pierce) and washed with three column volumes of wash buffer (50 mM Tris-HCl, 500 mM NaCl, 0.1 mM TCEP, 60 mM imidazole, 5% glycerol, pH 8.0). BirA was eluted in 1 ml fractions with elution buffer (50 mM Tris-HCl, 500 mM NaCl, 0.1 mM TCEP, 250 mM imidazole, 5% glycerol, pH 8.0). Fractions were subjected to SDS-PAGE to determine purity. Pure fractions were combined and dialyzed against storage buffer (50 mM Tris-HCl, 500 mM NaCl, 0.1 mM TCEP, 5% glycerol, pH 8.0). Aliquots were flash frozen and stored at −80°C.

The *B. subtilis* acetyl-CoA carboxylase biotin carboxyl carrier protein biotin attachment domain (AccB-86) was purified from *E. coli* strain BL21 (λ DE3) transformed with pSKH003. The strain was grown at 37°C in LB medium supplemented with kanamycin to an OD_600_ of 0.8 and induced by addition of IPTG to 1 mM for an additional 4 h at 30°C. The cells were centrifuged and resuspended in starting buffer (20 mM Tris-HCl, 1 mM NaCl, 0.1 mM TCEP, 5% glycerol, pH 8.0). The cells were lysed by passage through a French pressure cell. The lysates were centrifuged and the supernatant was subjected to 60% isopropanol precipitation followed by anion exchange chromatography using a HiTrap Q FF column (GE Healthcare) and fast liquid chromatography (AKTA) [37], [38].

The *B. subtilis* pyruvate carboxylase biotin attachment domain (PyC-77) was purified *E. coli* strain BL21 (λ DE3) transformed with pSKH004. The strain was grown at 37°C in LB medium supplemented with kanamycin to an OD_600_ of 0.8 and induced by addition of IPTG to 1 mM for an additional 4 h at 30°C. The cell were harvested by centrifugation and resuspended in starting buffer (50 mM sodium acetate, 100 mM NaCl, 0.1 mM TCEP, 5% glycerol, pH 4.9). The cells were lysed by passage though a French pressure cell and centrifuged. The supernatant was subjected to cation exchange chromatography using HiTrap SP FF column (GE Healthcare) and fast liquid chromatography (AKTA). PyC-77 was eluted by an NaCl gradient. Fractions were subjected to SDS-PAGE and fractions containing PyC-77 were combined and dialyzed against 50 mM 2-[4-(2-hydroxyethyl)piperazin-1-yl]ethanesulfonic acid (HEPES) buffer (pH 7.5) containing 500 mM NaCl and 0.1 mM TCEP. To further purify PyC-77 the protein was subjected to Superdex-75 size exclusion chromatography. Eluted fractions were analyzed by SDS-PAGE to determine purity. Fractions containing pure PyC-77 were combined, dialyzed against 50 mM HEPES buffer (pH 7.5) containing 500 mM NaCl, 0.1 mM TCEP, and 5% glycerol, flash frozen and stored at −80°C.

Plasmid pQC026C a derivative of vector pET28b encoding a terminal hexahistidine tagged BLAP was transformed into *E. coli* strain BL21 (λ DE3). The strain was grown in LB medium to an OD_600_ of 0.8 and induced by addition of IPTG to 1 mM for an additional 4 h. BLAP was purified by Ni^+2^ affinity chromatography as previously described [23]. The C-terminal hexahistidine tagged Δ2-65 BirA was purified as previously described [17].

### Electrophoretic Mobility Shift Assays (EMSA) of DNA binding

The predicted *B. subtilis* BirA binding sites upstream of the coding sequences of *bioO*, *yhfU* and *yuiG* were PCR amplified from *B. subtilis* 168 genomic DNA with primers SKH014 and SKH015, SKH016 and SKH017 and SKH018 and SKH019, respectively. A DNA fragment containing one half-site of *B. subtilis bioO* was similarly amplified with primers SKH034 and SKH035. *E. coli bioO* was amplified with primers SKH026 and SKH027 from MG1655 genomic DNA. Negative control DNA (*blap*) was amplified from *B. subtilis* 168 genomic DNA with primers SKH028 and SKH029. All DNA fragments were 125 bp in length. The PCR products were sized on a 1.8% agarose gel and purified using a QIAquick PCR Purification Kit (Qiagen). DNA concentrations were determined at OD_260_ by using a NanoDrop 2000c. Purified BirA was incubated with the small nucleophile hydroxylamine at neutral pH to cleave any Bio-5′-AMP bound in the active site [39] and then dialyzed against storage buffer. The reaction contained 50 mM Tris-HCl (pH 8.0), 1 mM EDTA, 50 mM NaCl, 10% glycerol, 40 nM DNA and various concentrations of BirA (500 nM, 250 nM, 125 nM, 62.5 nM, 31.25 nM, 15.6 nM), 1 mM ATP, 1 mM MgCl_2_, and 1 µM biotin [36]. The binding reactions were incubated at room temperature for 30 min and then loaded into a 6% DNA retardation gel (Invitrogen). The gel was run in 0.5X TBE at 100 V for 85 min. The gel was stained with SYBR Green I nucleic acid gel stain (Invitrogen) and visualized using Bio-Rad Chemidoc XRS and Quantity One software.

### Chemical Cross-linking of BirA

Purified BirA was dialyzed against 50 mM HEPES buffer (pH 7.5) containing 500 mM NaCl, 0.1 mM TCEP and 5% glycerol. BirA (30 µM) was incubated with or without biotin (0.1 mM) and ATP (0.1 mM) at room temperature for 30 min. Various concentrations of ethylene glycol bis[succinimidylsuccinate] (EGS) (0.125, 0.25, 0.375, 0.5 mM) were added and incubated at room temperature for 30 min. SDS-loading dye was added and samples were heated to 99°C for 5 min. Samples were loaded on 4–20% gradient SDS polyacrylamide gels (Bio-Rad) and run at 110 v for 1 h.

### Mass Spectrometry

Purified acceptor proteins AccB-86, PyC-77, and BLAP (10 µM), with or without incubation with BirA (0.5 µM), ATP (3 mM), and biotin (250 µM), were dialysed against 2 mM ammonium acetate, dried under a stream of nitrogen and subjected to electrospray mass spectrometric analysis [38].

### Bio-5′-AMP Synthesis Assays

The assays contained 50 mM Tris-HCl buffer (pH 8.0), 5.5 mM MgCl_2_, 100 mM KCl, 0.1 mM TCEP, 10 µM ATP, 25 µM biotin, 2.5 µM BirA, 0.1 µM [α-^32^P]ATP and with or without 50 µM AccB-86, PyC-77, or BLAP for a total reaction mixture of 20 µl [17], [36]. The reaction mixtures were incubated at room temperature for 30 min. A portion of each reaction mixture (1 µl) was spotted on cellulose thin-layer chromatography (TLC) plates and developed in isobutyric acid-NH_4_OH-water (66∶1∶33) [40]. The thin-layer chromatograms were dried for 10 h and exposed to a phosphorimaging screen and visualized using a Fujifilm FLA-3000 Phosphor Imager and Fujifilm Image Gauge software.

### β-Galactosidase Assays

Cultures were grown overnight in defined medium containing 1.6 nM biotin. The cultures were diluted to an OD_595_ of 0.2 in defined media containing various concentrations of biotin (1.6, 4, 20, 40, 80, 400 nM, 4 µM) and grown to OD_595_ of 0.8 and induced with 1 mM IPTG for an additional 2 h. β-Galactosidase activity was determined as described by Harwood and Cutting for *Bacillus* following permeabilization with lysozyme [26].

## Results

### *B. subtilis* BirA is a Group II BPL that binds three operator sites

**Figure 3:**
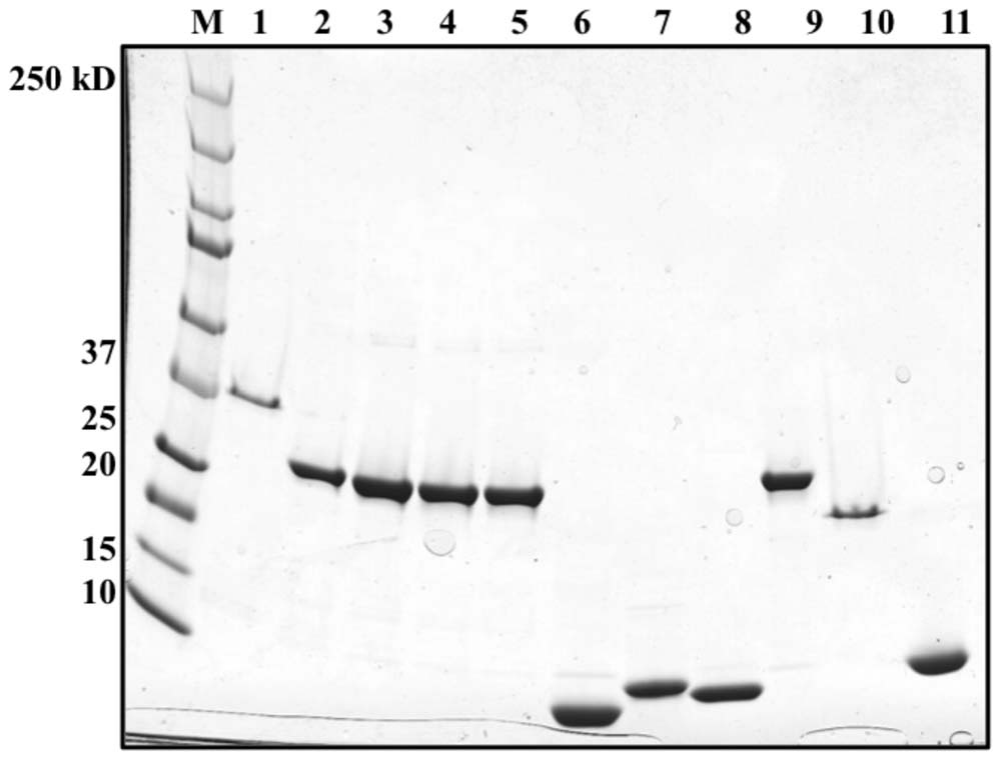
Purification of the wild type and N-terminal deletion BirA proteins and the biotin acceptor proteins. The proteins were purified as described in Materials and Methods and subjected to SDS-electrophoresis on a 4–20% polyacrylamide gel. M: molecular weight standards (Precision Plus Protein Standard Kaleidoscope from BioRad). Lane 1: *B. subtilis* N-terminally hexahistidine-tagged BirA (38.9 kDa). Lanes 2-5. *B. subtilis* N-terminally hexahistidine-tagged Δ2-63 BirA (31.8 kDa), Δ2-65 BirA (31.6 kDa), Δ2-74 BirA (30.4 kDa) and Δ1-81 BirA (29.7 kDa), respectively. Lanes 6-11 are the *B. subtilis* acceptor proteins AccB-86 (9.4 kDa), PyC-77 (8.3 kDa) and biotin lipoyl attachment protein (BLAP) (8.73 kDa). Lane 10 is *E. coli* C-terminal hexahistidine-tagged BirA. Lane 11 is *E. coli* C-terminal hexahistidine tagged Δ2-65 BirA (29.18 kDa) and lane 11 is *E. coli* AccB-87.

**Figure 4:**
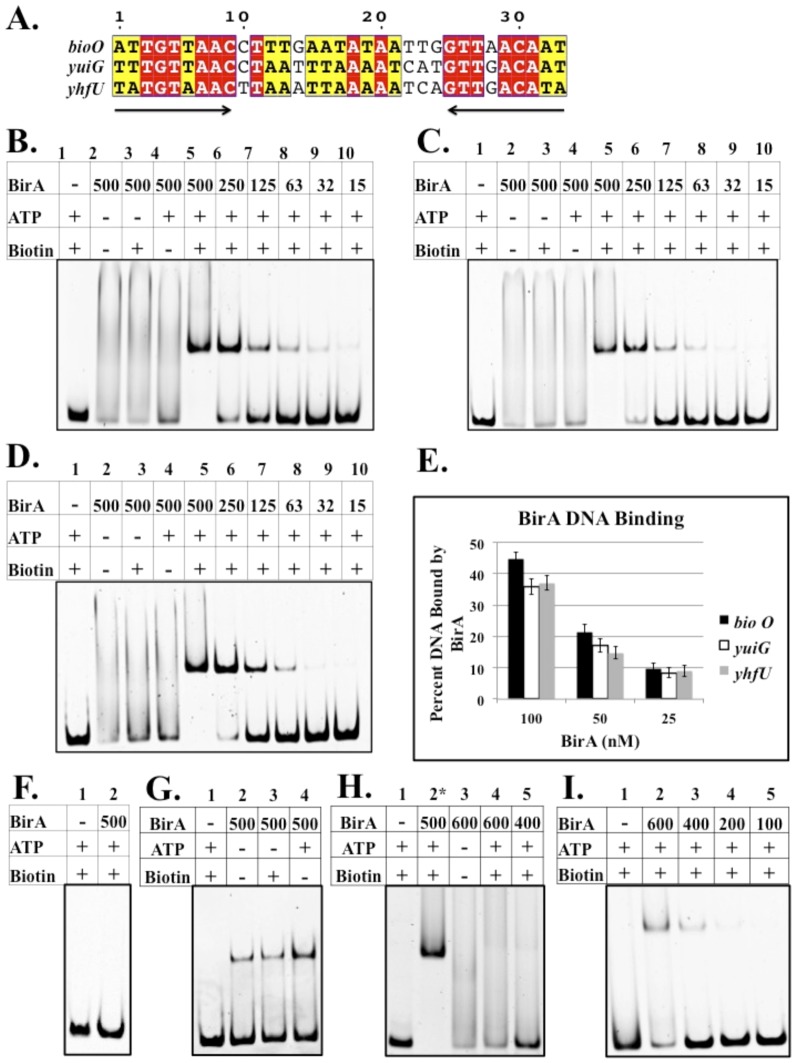
Sequence alignments of *B. subtilis* BirA DNA binding sites and electrophoretic mobility shift assay of DNA binding by BirA. *A. B. subtilis* has three predicted BirA DNA binding sites: 5′ UTR of the *bioWAFDBI* operon, 5′ UTR of *yuiG*, and 5′ UTR of the *yhfUTS* operon. Conserved residues are highlighted in red and similar residues are highlighted in yellow. *B C* and *D. B. subtilis* BirA binding to *bioO,* the *yuiG* operator and the *yhfU* operator, respectively. Note that only in the presence of biotin and ATP is binding observed. *E.* Quantitation of DNA binding by BirA (Quantity One software). The results show the average of three independent experiments, and the error bars denote standard error of the mean. *F.* BirA binding to non-operator DNA (a 125 bp internal fragment of the *yngHB* gene that encodes BLAP). *G.* BirA binding to *bioO* without hydroxylamine treatment. Bio-5′-AMP accumulates in the active site during expression in *E. coli* and survives purification of BirA. *H. B. subtilis* BirA binding to a half site of the inverted repeat of *B. subtilis bioO.* Note lane 2 is positive control full-length *bioO. I. B. subtilis* BirA binding to *E. coli bioO*. A collection of all putative BirA binding sites in diverse bacteria can be found in the RegPrecise database (http://regprecise.lbl.gov/RegPrecise/).

To test the relative affinities of the predicted *B. subtilis* BirA binding sites we purified the protein (Fig. 3) and performed electrophoretic mobility shift assays (EMSAs) on DNA fragments containing the three sites (Fig. 4A). Full dependence of binding on ATP and biotin required treatment of the protein with neutral hydroxylamine to remove Bio-5′-AMP accumulated in the active site during expression in *E. coli*. With the treated BirA binding of the biotin biosynthetic operator (*bioO*) was observed only in the presence of both biotin and ATP (Fig. 4B). BirA also bound the *yhfU* and *yuiG* operators only in the presence of biotin and ATP (Fig. 4C, D). Although the three binding sites have slightly different DNA sequences (Fig. 4A), analysis of binding over a range of BirA concentrations showed that the three sites had very similar binding affinities (Fig. 4E). *B. subtilis* BirA failed to show non-specific DNA binding (Fig. 4F) as assayed by use of a fragment from the coding sequence of the *yngHB* gene. BirA preparations that had not undergone hydroxylamine treatment showed some interaction with *bioO* in the absence of biotin and ATP (Fig. 4G). BirA did not interact with a site that was composed of only one of the *B. subtilis bioO* inverted repeats suggesting that the form of BirA active in DNA binding is a dimer (Fig. 4H). *B. subtilis* BirA interacted only very weakly with the *E. coli bioO* DNA site (Fig 4I).

### *B. subtilis* BirA biotinylates three cognate proteins and the reactions proceed via Bio-5′-AMP

**Figure 5:**
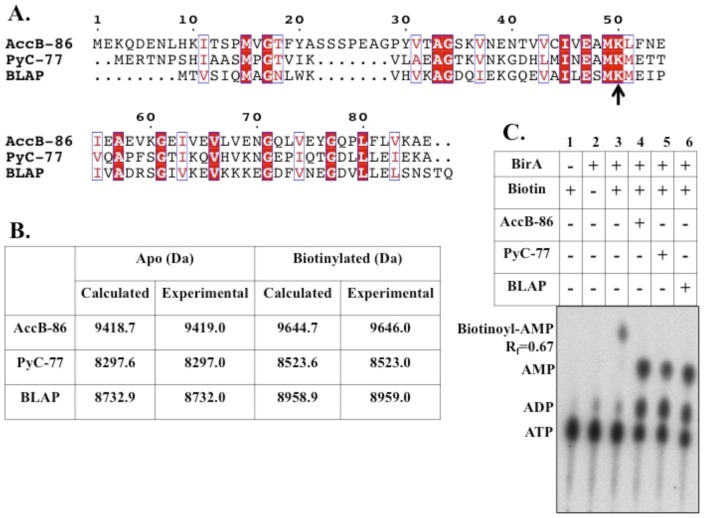
*In vitro* biotinylation of the *B. subtilis* biotin acceptor proteins. ***A.*** Sequence alignment of the *B. subtilis* biotinylated proteins. Conserved residues are in white text and highlighted in red and similar residues are in red text and boxed in blue. The black arrow indicates the conserved lysine residue that becomes biotinylated. ***B.*** Mass spectrometry values for purified acceptor proteins AccB-86, PyC-77, and BLAP. ***C.*** Thin layer chromatographic analysis of *B. subtilis* BirA ligase reaction: synthesis of Bio-5′-AMP and transfer of biotin to AccB-86, PyC-77, and BLAP.

Each of the acceptor proteins, AccB-86, PyC-77 and BLAP (Fig. 5A), was purified after high-level expression in *E. coli* (Fig. 3). In the first two cases the N-terminal halves of the proteins were deleted to avoid protein aggregation during purification (the deleted segments are responsible for interaction with other proteins of the enzyme complexes and play no role in biotinylation). Electrospray ionization mass spectrometry results matched the theoretical masses of the apo forms of the three proteins and showed that the preparations were free of the biotinylated forms (Fig. 5B). When the apo forms of AccB-86, PyC-77 and BLAP were incubated with ATP, biotin and purified *B. subtilis* BirA and subsequently analyzed by mass spectrometry, mass values very similar to the theoretical values for biotinylated forms of all three acceptor protein were obtained (Fig. 5B). In the presence of α-^32^P-labeled ATP and biotin *B. subtilis* BirA formed labeled Bio-5′-AMP. Upon addition of any of the three acceptor proteins (AccB-86, PyC-77, or BLAP) the Bio-5′-AMP intermediate was no longer detected and AMP accumulated which indicated transfer of biotin to each of the acceptor proteins (Fig. 5C). In conclusion, we have experimentally verified the bioinformatic analyses of the genes regulated by *B. subtilis* BirA and the proteins modified by its ligase activity.

### *B. subtilis* BirA does not require the N-terminal DNA binding domain for normal ligase activity

The *in vitro* data presented above indicate that *B. subtilis* BirA behaves in a manner that strongly parallels that of *E. coli* BirA despite the low sequence similarity of the two proteins. However, *E. coli* BirA has recently been shown to undergo extensive inter-domain communication that is required for full ligase activity [17]. These observations confirm and extend those of Xu and Beckett [16] and demonstrate that the *E. coli* BirA N-terminal domain plays a role in organizing the active site of BirA [17]. Specifically, the wing of the winged HTH structure interacts with the ligase active site biotin binding loop and acts to organize the active site to give high affinity binding of biotin and Bio-5′-AMP [17].

**Figure 6:**
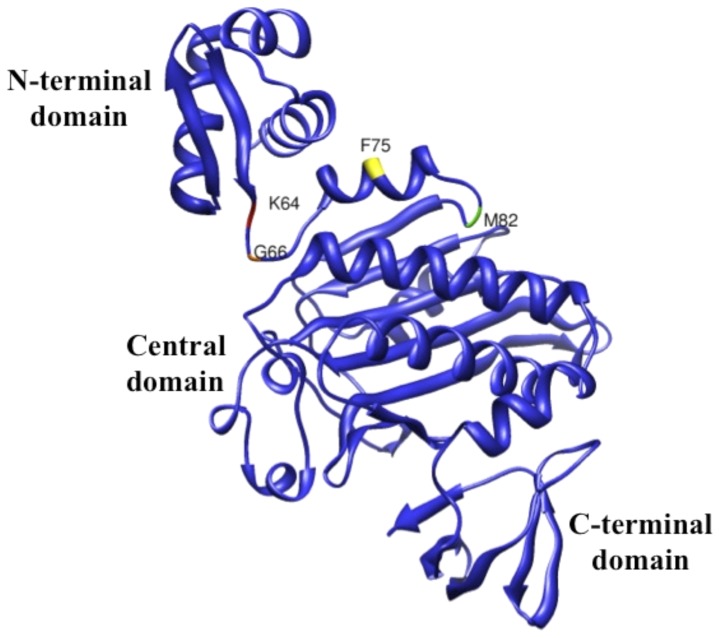
Model of *B. subtilis* BirA based on the *S. aureus* BirA crystal structure (PDB 4DQ2) [7]. The UCSF Chimera package [31] was used to create the image. Residues corresponding to the N-terminal deletion end points are given. Modeled domains are indicated.

**Figure 7:**
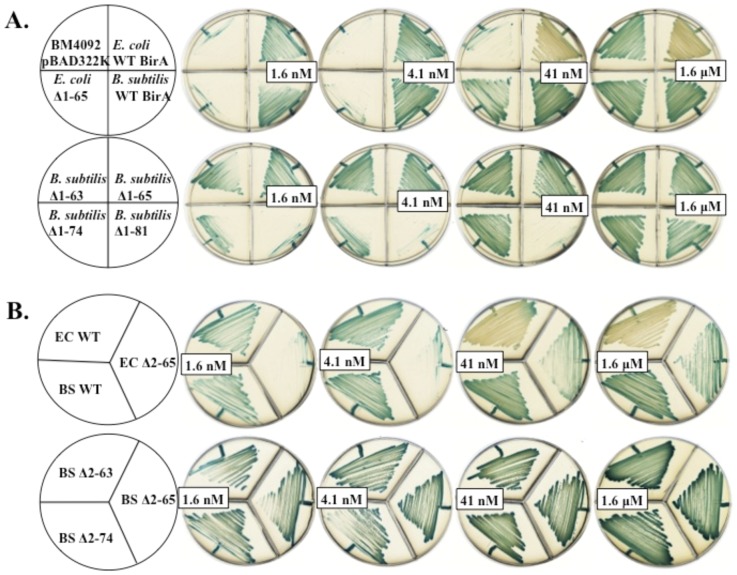
Complementation of *E. coli* strains by expression of the BirA N-terminal deletion proteins. ***A.*** Complementation of E. coli BirA mutant strain BM4092. ***B.*** Complementation of *E. coli ΔbirA* strain VC618. Strains were grown on M9 minimal medium containing different biotin concentrations (1.6 nM, 4.1 nM, 41 nM and 1.6 µM) and X-gal. The blue color indicates transcription of *bioF-lacZ* fusion. The white colonies indicate transcriptional repression of the biotin operon by BirA binding at *bioO*. Note that *B. subtilis* wild type BirA does not complement the regulatory function of *E. coli* BirA and thus gives blue colonies.

To determine if another Group II BPL, that of *B. subtilis*, requires such communication of the catalytic and N-terminal domains for full ligase activity, we constructed genes encoding several N-terminal *B. subtilis* BirA deletion proteins. The deletion endpoints were based on structural modeling of *B. subtilis* BirA based on the crystal structure of *S. aureus* BirA (PDB 4DQ2) [7] (Fig. 6). BirA deletions Δ2-63 and Δ2-65 eliminated the predicted N-terminal domain whereas BirA deletions Δ2-74 and Δ1-81 also cut into the predicted central catalytic domain. Complementation assays using the *E. coli birA1* mutant strain BM4092 and the *E. coli ΔbirA* deletion strain VC618 were used to test the ligase activities of the *B. subtilis* BirA N-terminally deleted proteins. These assays showed that upon expression of the *B. subtilis* Δ2-63 BirA, Δ2-65 BirA and wild type proteins in strain BM4092 all three BPLs supported growth equally well on medium containing 1.6 nM biotin (the minimal level allowing growth of *E. coli*) (Fig. 7A). These results indicated that the ligase activities of these two deletion proteins were essentially normal. In contrast, upon expression of the *E. coli* BirA lacking its amino terminus (Δ2-65 BirA), growth of the transformed strain required a biotin concentration that was 1000-fold greater. The complementation activities of the other two *B. subtilis* deletion proteins, BirA Δ2-74 and BirA Δ1-81 were either partially (BirA Δ2-74) or totally (BirA Δ1-81) compromised (Fig. 7A). To ensure that residual activity of the host BirA1 protein did not play a role in growth restoration we also performed complementation of the *ΔbirA E. coli* strain VC618 and obtained a similar complementation pattern (Fig. 7B).

**Figure 8:**
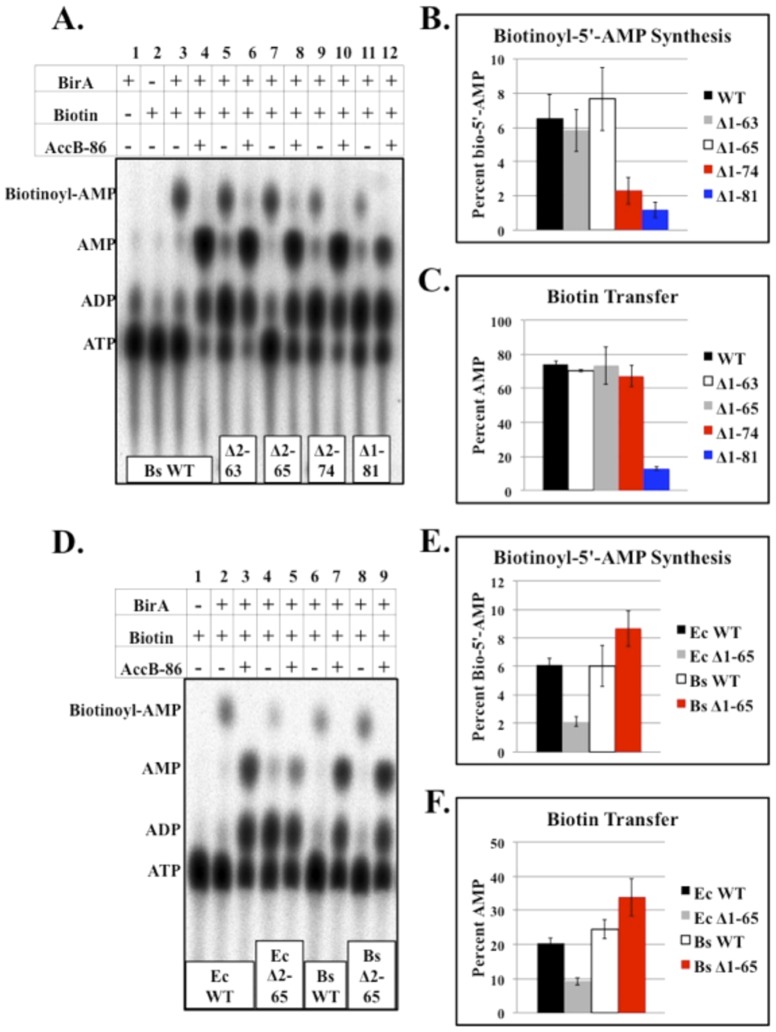
*In vitro* biotinylation analyses of the BirA N-terminal deletion proteins. ***A.*** Thin layer chromatographic analysis of wild type *B. subtilis* BirA and *B. subtilis* BirA N-terminal deletions with and without the addition of acceptor protein AccB-86. ***B.*** Quantitation of Bio-5′-AMP synthesis by wild type *B. subtilis* BirA and the *B. subtilis* BirA N-terminal deletion proteins. The results show the average of three independent experiments, and the error bars denote standard error of the mean. ***C.*** Quantitation of biotin transfer to AccB-86 by wild type *B. subtilis* BirA and *B. subtilis* BirA N-terminal deletions. The results show the average of three independent experiments, and the error bars denote standard error of the mean. ***D.*** Thin layer chromatographic analysis of wild type *E. coli* BirA, *E. coli* Δ2-65 BirA, wild type *B. subtilis* BirA, and *B. subtilis* Δ2-65 BirA. ***E.*** Quantitation of Bio-5′-AMP synthesis by wild type *E. coli* BirA, *E. coli* Δ2-65 BirA, wild type *B. subtilis* BirA, and *B. subtilis* Δ2-65 BirA. The results show the average of three independent experiments, and the error bars denote standard error of the mean. ***F.*** Quantitation of biotin transfer to *E. coli* AccB-87 or *B. subtilis* AccB-86 by wild type *E. coli* BirA, *E. coli* Δ2-65 BirA, wild type *B. subtilis* BirA and *B. subtilis* Δ2-65 BirA. The results show the average of three independent experiments, and the error bars denote standard error of the mean.

**Figure 9:**
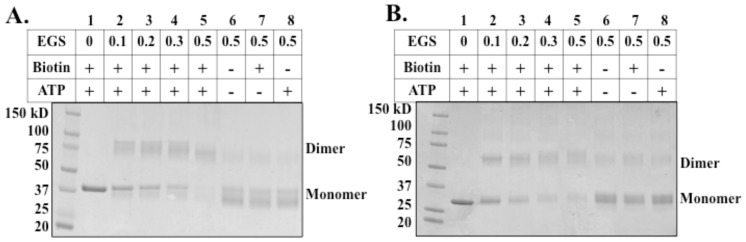
Chemical crosslinking of the *B. subtilis* wild type and Δ2-65 BirA proteins. ***A.*** Wild type BirA. ***B.*** The Δ2-65 BirA. Note that efficient dimer formation requires the presence of both biotin and ATP. The EGS concentrations are in mM.

The ligase activities of the purified *B. subtilis* BirA N-terminal deletion proteins (Fig. 3) were also tested by *in vitro* biotinylation assays. As expected from the complementation results the Δ2-63 and Δ2-65 BirA proteins showed Bio-5′-AMP synthesis and biotin transfer activities indistinguishable from those of the wild type protein (Fig. 8). In contrast the Bio-5′-AMP synthetic abilities of the Δ2-74 and Δ1-81 BirAs were significantly reduced relative to wild type BirA. However, the biotin transfer activity of the Δ2-74 BirA was comparable to wild type levels. Biotin transfer by the Δ1-81 BirA was significantly reduced compared to the wild type protein (Fig. 9). In agreement with prior work [16], [17] the *E. coli* Δ2-65 BirA was significantly reduced in Bio-5′-AMP synthesis and in biotin transfer (Fig. 8).

### *Bacillus subtilis* BirA dimerizes in the presence of biotin and ATP

Dimerization is required for DNA binding of *E. coli* BirA and dimerization is dependent on biotin and ATP binding. To determine the oligomerization state of *B. subtilis* BirA, purified BirA was incubated with EGS in the presence or absence of biotin and ATP. Chemical crosslinking indicated that BirA forms a dimer only in the presence of both biotin and ATP (Fig. 9A). Dimerization was also observed for the N-terminally truncated Δ2-65 BirA (Fig. 9B). Note that the characterized Group I BPLs can either be dimeric as is the *Pyrococcus horikoshii* protein [3], [41] or monomeric as are the *Mycobacterium tuberculosis* [4], *Aquifex aeolicus* [5] and *Propionibacterium freudenreichii* subsp. *shermanii* [42] BPLs.

### *Bacillus subtilis* BirA follows the *E.* coli BirA regulatory model

In *E. coli* regulation of the biotin biosynthetic gene transcription depends not only on the concentration of biotin but also the levels of unbiotinylated AccB [8]–[10]. This is an important attribute because biotin is only active in central metabolism when it is protein bound. Hence, the rate of biotin operon transcription is sensitive not only to the intracellular concentration of biotin, but also to the supply of the proteins to which the biotin must be attached. Moreover, accumulation of the unmodified protein increases the rate of biotin synthesis thus ensuring replacement of the biotin consumed in protein modification.

**Figure 10:**
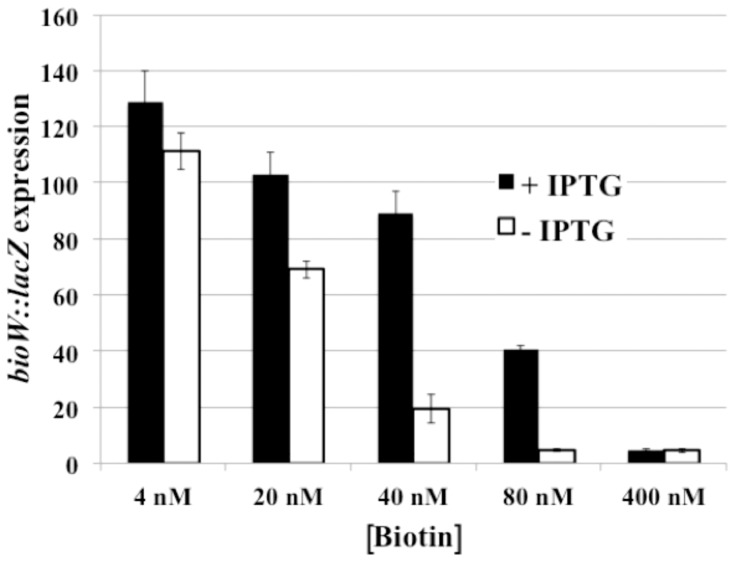
β-Galactosidase assays of the effect of AccB-86 levels on *bioO*-dependent transcription. The *bioW::lacZ* spac *accB-86* strain SKH002 was grown in defined medium supplemented with the indicated concentrations of biotin plus or minus IPTG addition to induce synthesis of the AccB-86 acceptor protein. The results are the average of three independent experiments and the error bars denote standard error of the mean.

To determine if this regulatory facet also applies to another Group II BPL, we constructed a *B. subtilis* strain that could be used to monitor regulation of biotin operon transcription upon alteration of the levels of the AccB-86 acceptor protein. This strain contains the *bioB141* mutation rendering it a biotin auxotroph, a *bioW-lacZ* transcriptional fusion and an ectopic IPTG-inducible gene encoding AccB-86. This strain was grown in chemically defined media containing various concentrations of biotin. Upon induction of AccB-86 expression with IPTG β-galactosidase assays showed that expression of the biotin operon was dereprepressed at biotin concentrations that normally cause repression (Fig. 10) in a manner similar to that seen in *E. coli* [8]-[10].

## Discussion

*B. subtilis* BirA complements the ligase activity of *E. coli* BirA mutant strain BM4092, but fails to complement the regulatory function of *E. coli* BirA. This latter result is not surprising since *E. coli* and *B. subtilis* BirAs bind different operator sequences and have different spacing of the palindromic elements. We have shown that *B. subtilis* BirA is a Group II BPL that binds equally well to the three operators predicted by others (Fig. 4). Binding is observed only in the presence of both biotin and ATP indicating that Bio-5′-AMP is the regulatory ligand. Interestingly, EMSA showed that *B. subtilis* BirA weakly binds *E. coli bioO* (Fig. 4I), suggesting some distant relationship between the operators. Similar to *E. coli*, dimerization of *B. subtilis* BirA occurs only in the presence of biotin and ATP, a further indication that Bio-5′-AMP is the regulatory ligand (Fig. 9A). The *B. subtilis* Δ2-65 BirA also formed dimers indicating that the N-terminal domain is not required for dimerization (Fig. 9B).

Deletions of the N-terminal DNA binding domain of *E. coli* BirA result in proteins having only very weak ligase activities indicating inter-domain interactions are required for full ligase activity [16], [17]. These interactions have been shown to occur between the wing of the winged HTH domain and the biotin-binding loop of the catalytic domain. Deletion of only the fourteen-residue wing has as drastic an effect as deletion of the entire N-terminal domain, but was largely restored by insertion of a foreign wing of similar structure [17]. Moreover, a mutation within the wing can restore function to proteins having mutant biotin binding loops [17].

Although the HTH domain of our modeled *B. subtilis* BirA structure includes a wing, the structure is not required for full ligase activity. Both the Δ2-63 and Δ2-65 BirAs lack the entire N-terminal domain but performed the ligase partial reactions as well as the wild type protein both *in vivo* and *in vitro* (Fig. 8). Deletions that entered the predicted catalytic core of the protein resulted in either compromised (Δ2-74 BirA) or highly defective (Δ1-81 BirA) proteins (Fig. 8). The Bio-5′-AMP synthetic ability of the Δ2-74 BirA was significantly decreased, although the protein transferred the biotin moiety normally suggesting that the putative α-helix near residue 74 may be involved in stabilizing biotin and ATP or Bio-5′-AMP binding. Although *B. subtilis* Δ1-81 BirA failed to replace the *E. coli* ligase *in vivo* (Fig. 7), it retained weak Bio-5′-AMP synthesis and biotin transfer activities (Fig. 8) suggesting that the loop predicted near residue 81 is required for efficient binding of biotin and ATP. These data indicate that unlike *E. coli*, *B. subtilis* BirA does not require an intact N-terminal DNA binding domain for full ligase activity. Instead, the first α-helix and loop of the modeled central domain seem to be important in binding biotin and ATP.

*E. coli* BirA is a highly dynamic protein as shown both by physical [43]–[45] and mutational [36] analyses. In the latter case mutations in the catalytic and C-terminal domains as well as in the HTH domain can result in super-repressor phenotypes. These proteins are BirAs that repress *bio* operon expression even at biotin concentrations that normally fail to repress transcription. Although *E. coli* BirA has been studied for over 30 years [40], [46] and four different crystal structures have been solved, the details of how the protein binds its operator site remain unknown. Despite much effort in several laboratories no diffraction grade crystals of the operator DNA with liganded *E. coli* BirA have been obtained. This could be due to competing interactions of the N-terminal domain with the catalytic domain and operator giving a mixture of molecular species that preclude crystallization. If so, the apparent lack of such interactions in *B. subtilis* BirA may allow crystallization of this protein with its operator DNA.

The BLAP protein was biotinylated by *B. subtilis* BirA *in vitro* suggesting that BLAP may be the biotin carboxyl carrier protein for a biotin-dependent enzyme. The genes that neighbor *yngHB*, the BLAP encoding gene, are annotated as propionyl-CoA carboxylase subunits. However, a recent report suggests that these genes may comprise a methylcrotonyl-CoA carboxylase involved in leucine degradation [47].
